# Incorporating Radar Frequency-Domain Deramping into Variational Shape-Based Scene Reconstruction: A Feasibility Study Using Active Contours

**DOI:** 10.3390/s25082451

**Published:** 2025-04-13

**Authors:** Alper Yildirim, Samuel Bignardi, Christopher F. Barnes, Anthony Joseph Yezzi

**Affiliations:** 1 Georgia Institute of Technology, School of Electrical and Computer Engineering, Atlanta, GA 30332, USA; chris.barnes@gatech.edu (C.F.B.);; 2 Department of Engineering and Geology, University “G. D’Annunzio” of Chieti-Pescara, 66110 Chieti, Italy; samuel.bignardi@unich.it; 3 UDA-TECHLAB Research Center, 66110 Chieti, Italy

**Keywords:** radar, shape reconstruction, noncoherent, inversion

## Abstract

Multi-view stereo techniques with traditional cameras have wide applications in robotics and computer vision for scene reconstruction. Their dependence on the visible spectrum, however, poses several limitations that radar sensing could overcome in obstructing conditions such as fog and smoke. We propose a new radar-based multi-view stereo method for scene reconstruction, which combines the power of multi-view stereo techniques with the advantages of radar sensing by extending upon our previous work in this direction, where we demonstrated a time-domain inversion approach by leveraging a set of independent radar echoes acquired at sparse locations to reconstruct the scene’s geometry. Here, we show how radar stretch processing can be incorporated into a similar geometric framework to leverage frequency-domain information. Our method fundamentally differs from classical radar imaging by utilizing an explicit geometric shape representation, allowing the imposition of shape priors and the ability to model visibility and occlusions, and a forward model based on the electric field strength density over the antenna range embedded within the deramped echo. An iterative scheme is then used to evolve an initial shape toward an optimal configuration to best explain the data. We conclude by showing the initial proof of concept for the success of this method through a set of simulated 2D experiments of increasing complexity.

## 1. Introduction

Computer vision systems are becoming increasingly essential in several scientific fields, especially robotics and machine perception. Understanding a scene and the shape of the objects therein is a major challenge in many surveillance, navigation, and segmentation approaches. In this context, inferring the geometry of a scene using visual cues from camera images acquired at sparse locations, also known as the “stereo reconstruction”, is a well-established approach.Variational methods fall into this class of applications, where the reconstruction problem is cast into a classic inversion scheme using a 2D/3D geometric model (i.e., a curve or surface), and this model is then evolved to outline the object of interest [1].

On the one hand, the mathematical framework of shape reconstruction from images is well understood. On the other hand, stereo vision is inherently limited by its reliance on information within the visible spectrum and becomes unreliable in low ambient light or in the presence of obstructing factors such as rain, fog, or smoke. The integration of radar sensing within such inversion frameworks would be highly beneficial, as radars can operate effectively at night and can probe scenes even when adverse weather conditions render the optical alternatives ineffective [2].

In order to probe the investigated scene, classical radar imaging techniques leverage actively generated signals, often linear frequency-modulated (LFM) chirps, emitted by a moving antenna. The back-scattered echoes recorded at different locations on the antenna flight path are then combined (i.e., coherently summed) to generate a high-resolution image of the area of interest. This technique is known as Synthetic Aperture Radar (SAR) imaging [3,4,5]. It should be noted that coherent summation requires the synchronization of signals and the careful control of the antenna’s position along the flight path. In addition, processing techniques typically rely on regular sampling in space. As such, SAR applications are restricted to linear or circular antenna paths, and data acquisition becomes more complex as the signal frequency increases.

SAR has also been used in connection with additional post-processing techniques to retrieve the geometric properties of scenes. Interferometry and differential interferometry [6,7], for example, are routinely used to obtain land topography or to monitor changes in the scene over time. SAR technology has been extended to three-dimensional imaging applications, where the scene is modeled as a 3D reflectivity function with values computed from measurements [8,9]. Complementing these tomographic approaches, some near-field techniques have also been proposed for 3D SAR [10].

SAR does not offer directly retrievable representations of shapes within the scene, and shape reconstruction is typically achieved by post-processing the radar imaging products, often leveraging computer vision techniques originally designed for optical images.

Although image formation requires simple but rigid antenna configurations, a reconstruction where both radar and camera data are collected jointly at sparse locations and are capable of combining both sources of information within the same mathematical framework would be highly desirable. However, before tackling such a joint inversion task, we first demonstrate that radars can be used successfully by themselves, even if less practically, in a framework of shape reconstruction. In principle, embedding radar signals into an inversion scheme can be achieved by constructing an energy functional (or cost function) using a least-squares approach aimed at minimizing the differences between recorded signals and their simulated counterparts (with the latter computed from a suitable reference model). This methodology is commonly known as Full Waveform Inversion (FWI) and has demonstrated a good degree of success with acoustic signals both in geophysics, medical applications, and non-destructive testing [11,12,13], where the model being optimized typically consists of the distribution of sought physical properties within the cells (or at the nodes) of a discretized volume. However, despite its sound mathematical foundation, practical FWI applications suffer from the “cycle-skipping” phenomenon [11], which becomes increasingly severe at higher frequencies. Cycle skipping is already a significant limitation when dealing with acoustic frequencies, and it would render radar-based applications, which typically operate at frequencies of the order of MHz or GHz, effectively unfeasible. In this context, Yildirim et al., 2018 [14] and Bignardi et al., 2021 [15] demonstrated that it is possible to leverage variational methods to build an inversion framework in all aspects analogous to stereo reconstruction from images, but radar signals can be leveraged instead. Even when the return signal is demodulated, their inclusion in a least-squares form leads to optimization strategies affected by a large number of local minima. For this reason, shape inversion has rarely been used with this kind of data. Nevertheless, Bignardi et al., 2021 [15] demonstrated that shape reconstruction can still be achieved after applying a suitable preprocessing strategy (specifically designed to mitigate the cycle-skipping problem), showing that radar-based inversion is robust enough to enable shape reconstruction, even by itself.

Therefore, in contrast to traditional radar imaging, where the focus is to create 2D/3D images (a grid of discrete pixels/voxels), we tackle the reconstruction problem by inverting the high-frequency radar signal back-reflected from the scene using a deformable shape model. In particular, while Bignardi et al. [15] formulated the inversion of pulse-compressed signals in the time domain and demonstrated that the approach is suitable for shape reconstruction using the level-set method as an implicit representation of the scene geometry [16], we investigate here the shape reconstruction problem from deramped (i.e., stretch-processed) radar echoes in the frequency domain. We obtain a variational approach where we seek to decrease an energy functional designed to be minimal when the shape’s radar response is most similar to the input data. During minimization, our initial shape will evolve continuously until the desired geometry is found.

Despite the different formulations, several of the results from our previous work [15,16] are immediately applicable here as well. In our formulation, the inversion problem relies on the capability of optimizing the geometric shape and location of a scene so that the amount of power back-reflected toward the receiving antennas from the portions of the scene sitting at specific distances reproduces the data. In carrying this out, the problem becomes dependent on the amplitude of the signals rather than on the Doppler shift (i.e., the signal phases). Unfortunately however, the pulse compression approach [15] cannot be straightforwardly carried over to the frequency domain but requires a change in the preprocessing strategy. We have found that leveraging stretch processing can be successful, as it allows one to cast the back-scattered time series (i.e., time-dependent information) in a range-dependent electric field strength profile over a certain range. Our approach depends mainly on the signal strength over the range for each measurement, and we do not make use of the phase relations across different measurements, which makes our method noncoherent. The immediate advantages of combining a noncoherent inversion approach with an explicit-shape model-based inversion are (1) the ability to handle sparse measurements and independence from hardware precision and tolerance considerations (coherence becomes harder to implement as the signal frequencies increase) and (2) the natural ability to handle visibility and occlusions [17,18].

Since some of these aspects were shared and discussed in depth in Bignardi et al. (2021, 2023) [15,16], where a purely time domain approach was taken using pulse compression, the goal of this paper is to utilize the stretch processing technique to lay the foundation of a frequency-domain formulation for shape reconstruction within a similar explicit geometric framework. We will show, in a simplified 2D context at this initial stage, that active contours and the level-set method (LSM) can be leveraged for shape evolution in conjunction with deramping techniques and will therefore be an ideal environment for further three-dimensional development.

In the following, Section 2 describes how we formulate the cost functional for our inversion using preprocessed data and details how we compute the observable quantities in the frequency domain. In particular, while the mathematical framework developed here is valid for general three-dimensional problems, since we already know that this class of problems can be easily formulated in three dimensions [15], we consider two-dimensional geometries for ease of implementation and focus our attention on assessing the success of the frequency-domain reconstruction instead. Section 3 will demonstrate some example reconstructions in which the geometry of the scene is represented by a polygonal object. Finally, we discuss our findings and conclusions in Section 4.

## 2. Materials and Methods

**Figure 1:**
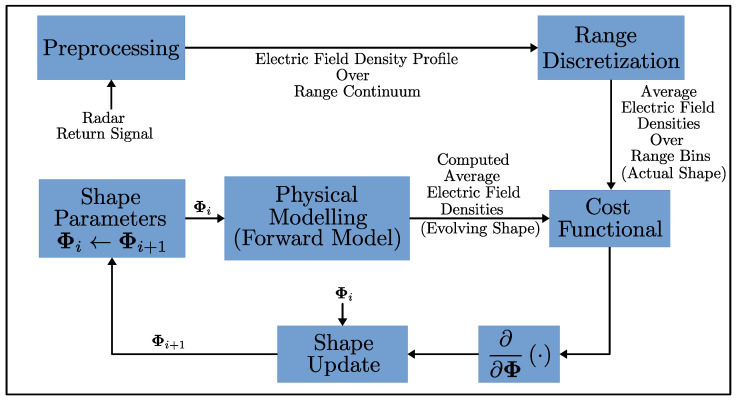
Shape inversion algorithm flow chart for our method.

We consider a scene/object that we want to reconstruct, and it is probed by a set of antennas arbitrarily located around the scene. We assume that the antenna locations are known, and these antennas are probing the scene by transmitting linear frequency-modulated (LFM) chirp pulses and collecting the echos. These echos are then deramped, which provides the key observable that used in our method: that is, an electric field strength density profile over the antenna range. This is possible from the fact that all reflections coming from a certain range are represented by a unique frequency signature in the deramped signal, and thus, the spectral analysis of the deramped signal yields, on average, the amount of electric field strength reflected from a given range. We will briefly discuss why extracting such information from the echos is feasible and formulate our forward model based on this observable quantity. As a result, our forward model, given an antenna location and a shape, will generate a vector of average electric field strengths over a range decomposition of the space centered around the antenna. Our inverse model will then be responsible for comparing these vectors of electric field strengths to those obtained from our simulated measurements (output of the forward model for the actual scene we do not know) and evolving our shape model over iterations to match our deformable shape model to the actual scene. For ease of implementation, we will use a polygonal shape representation for the scene. Figure 1 shows an overview of our shape reconstruction method, while the following subsections will detail several aspects of the different components listed therein.

### 2.1. Preprocessing

Since our shape estimation scheme relies on iteratively evolving a shape model to match the actual scene, careful design of the cost functional is critical. This is particularly challenging for radar sensing, as our primary information sources are signals oscillating at high frequencies. Incorporating radar data within a least-squares energy functional presents a significant challenge, as the resulting functional would be affected by an intractable number of local minima [15]. Consequently, a simplistic design for the cost functional would transfer this high-frequency behavior into the parameter space (shape), populating our functional with unwanted local minima due to cycle-skipping phenomena [11]. For an iterative algorithm that depends on gradient information, this complication renders the approach impractical, as gradient information will drive the shape’s evolution toward the nearest local minimum. This concern is especially relevant for applications like ours that utilize high-bandwidth waveforms. To address this issue, we propose a novel preprocessing method for our return signal that enables us to design our cost functional using information independent of the high-frequency waveform structure. Ideally, the information (observable) to be extracted from the radar signal should possess the following three properties for our method to work:

- It should maximize the retention of the geometric information contained within the radar echo.
- It should be as decoupled from the high-frequency oscillatory structure of the carrier waveform as possible.
- It should exhibit smooth variations with respect to shape geometry, ensuring gradient information for guiding evolving shape models to the actual shape.

### 2.2. Extraction of Field Strength Profile over Range

Radar measurements comprise back-reflected returns from individual scatterers within the scene that interfere both constructively and destructively to form our observed signal. Consequently, scene shape information is embedded in the radar’s return signal in a highly convoluted manner, making the raw data unsuitable for our applications. Furthermore, significant hardware constraints must be addressed, as most radar systems operate at frequencies substantially exceeding the sampling capabilities of even advanced analog-to-digital converters (ADC) [19]. Although demodulation techniques can partially mitigate sampling requirements, challenges persist when processing high-bandwidth signals. However, since a geometric object placed before the radar spans a continuum of range values, the resulting echo represents a composite of return signals with varying time delays. To address these challenges, we propose a two-step preprocessing methodology to decompose the return signal into its range-dependent components.

The first step uses the established deramping technique (also termed stretch processing [20]). This approach involves mixing the radar return signal with a heterodyne signal—a time-delayed replica of the transmitted signal. This process generates a new signal with substantially reduced frequency components, thereby significantly lowering sampling requirements. Additionally, when the transmitted signal is a linear frequency-modulated (LFM) pulse, this technique effectively produces a range decomposition of the radar echo. From a mathematical perspective, for a scene scatterer with a range (round-trip distance) of $D_{r}$ relative to a transmitter–receiver pair, the stretch processor output (excluding pulse windowing) is expressed as follows:

(1) $$h\left(t\right)=A_{h}e^{i2\pi f_{c}\left(t_{r}-t_{h}\right)}e^{-i2\pi \alpha \left(t_{r}^{2}-t_{h}^{2}\right)}e^{i4\pi \alpha \left(t_{r}-t_{h}\right)t}$$

where $D_{r}=ct_{r}$; $A_{h}$ represents the strength of the return signal; $f_{c}$ denotes the center frequency of the transmitted pulse; α is the chirp rate; $t_{r}$ and $t_{h}$ are the delay of the echo and heterodyne signal (delayed replica of the transmitter signal), respectively. The equation reveals that only the final exponential term exhibits a time dependence with a frequency of $2\alpha \left(t_{r}-t_{h}\right)$. Since $t_{r}$ and $D_{r}$ are related through the speed of light *c*, we observe a frequency value that varies linearly with range.

In our case, a geometric shape with its visible portion spanning a continuum of ranges results in a deramped signal that contains a continuum of frequencies within interval $\left[f_{min},f_{max}\right]$. This interval is determined by the minimum and maximum range values ($\left[D_{min},D_{max}\right]$) of the visible portion of the object relative to the corresponding antenna pair. Consequently, the frequency spectrum of the deramped signal encodes the distribution of the electric-field strength density along the shape’s range profile.

**Figure 2:**
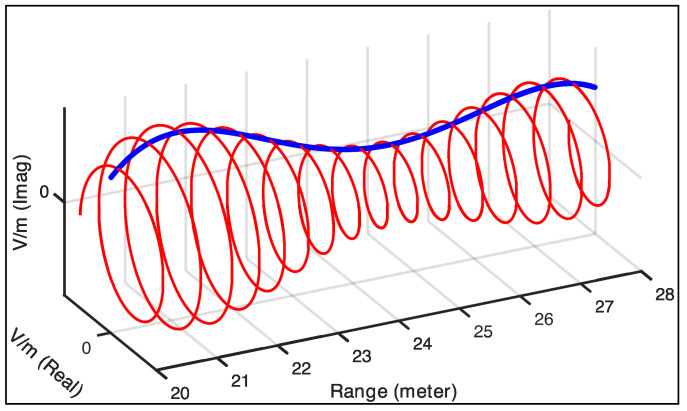
Depiction of the frequency (range) spectrum of stretch-processed echoes between $\left[D_{min},D_{max}\right]$ assuming no windowing applied in the time domain. The range interval in this scenario is $\left[20\;m,28\;m\right]$. The red curve is the decomposition of the deramped signal, and the blue curve is the envelope.

It is important to recognize that even though the density of the electric field’s strength reflected from a specific range is represented by $A_{h}$ in Equation (1), we also have two new terms in the equation (second and third terms that are carrier-dependent and residual video phase terms) that introduce complex phase expressions that vary with time delay (or range) and manifest themselves in the frequency spectrum of the stretch-processed signal. Figure 2 illustrates a typical frequency spectrum of a stretch-processed signal between the range interval $\left[D_{min},D_{max}\right]$ assuming that there is no windowing in the time domain. Our primary interest lies in the electric field’s strength density ($A_{h}$), as this component carries the geometric information relevant to our method.

Notably, our method does not require estimating the continuous profile, as we utilize a discretized version of the electric field strength density over range bins. Thus, our objective is to estimate a set of average electric field strength values across specified ranges using time samples of the stretch-processed signal. Although we have proposed an approach for this estimation [21], it has not been implemented in the current paper. Similar decomposition techniques (combining stretch processing with discrete Fourier transforms) have been established in the SAR literature, such as polar formatting [22], demonstrating the viability of range decomposition from stretch-processed radar returns. Furthermore, compensation methods for the phase terms in Equation (1) have also been studied [23].

### 2.3. Forward Model

**Figure 3:**
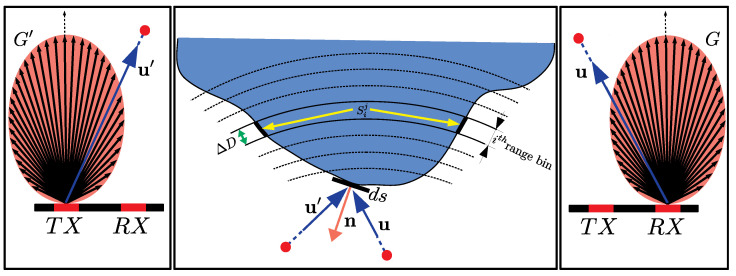
Illustration of our forward model. Directional antennas serve as transmitter (TX) and receiver (RX). The unit vectors $u^{\prime }$ and u represent directions from TX and RX relative to the scene, respectively, while n denotes the unit normal vector of a point scatterer. The integration domain for the *i*-th range bin for the *j*-th antenna pair is represented by $S_{i}^{j}$, comprising two curve segments (under the assumption that they are visible to the antenna pair). The parameter ΔD indicates the width of the range bin of interest.

Our forward model is one of the core components of our inversion, and it is used to simulate the radar response of a given shape. The inputs to this component are the evolving shape estimate C, the associated reflectivity that rules the strength of the scattered signal, and the configuration of the transmitting/receiving antenna pair (TX/RX). For simplicity, let us consider just one transmitter–receiver pair with known locations. The scene C is partitioned into range bins. The part of the scene with a range that falls within a specific bin determines the value of our observable quantity H(C) for that bin. The range bins can be visualized as a set of ellipsoids with foci that coincide with the locations of TX and RX (Figure 3). For a given range bin, the value of the corresponding sample H(C) is obtained by integrating over the portion of the surface included within the bin, which is also visible from both the transmitting and receiving antennas.

In our simplified 2D development, we model the infinite-small electric field strength on the receiver induced by an infinitely small length scatterer ds located at x, which is described as follows:

(2) $$dH=Q\left(x,n\right)ds,$$

where the scatterer unit’s normal is denoted with n. In practice, for computation purposes, we adopt several assumptions, namely the following: (1) the transmitting and receiving antennas are directional; (2) the scene consists of a continuum of ideal point scatterers that behave as Lambertian reflectors; (3) multi-path scattering is neglected. Consequently, $Q\left(x,n\right)$ becomes the following:

(3) $$Q\left(x,n\right)=\frac{G^{\prime }\left(x\right)G\left(x\right)\sqrt{-u^{\prime }\left(x\right)\cdot n}\sqrt{-u\left(x\right)\cdot n}}{R^{\prime }\left(x\right)R\left(x\right)}$$

where $u^{\prime }$ and u are unit vectors from TX and RX pointing to the scene, $R^{\prime }$ and *R* are the corresponding distances, and $G^{\prime }$ and *G* are antenna directivity multipliers for transmitted/received signal strengths in the direction of point x with respect to the antenna’s normal. In this paper, we will use the term “antenna gains” to refer to these quantities, though it should be noted that within our model framework, these values are characterized in terms of the electric field strength rather than the conventional definition that expresses them in terms of transmitted/received power.

In summary, given an estimate of the shape of the scene C and the known locations of TX and RX, the radar signal is emitted by TX probes in the scene and is scattered back toward receiver RX. Our observable quantity H(C), while being a function of the frequency (discretized in bins), still retains information on the depth (the increasing frequency is associated with increasing ranges), at which different portions of the scene are encountered. In addition, the strength of the electric field measured from a specific depth carries information about the physical aspects and the size of the portion of the scene included within the corresponding bin. The latter is computed as follows:

(4) $$H_{i}^{j}=\frac{1}{\Delta D}\int_{S_{i}^{j}}\chi \left(x\right)Q\left(x,n\right)ds$$

where ΔD is the width of the range bin, and $S_{i}^{j}$ is the set of scatterers on the shape boundary contained within the *i*-th range bin associated with the *j*-th antenna pair. Finally, χ(x) is an indicator function that takes into account whether the scene at x is hidden either from the emitter or the receiver’s point of view. In this way, our model explicitly accounts for visibility and self-occlusions (which is not the case for radar imaging). The present model retains the same properties both in three dimensions, where the shape is a surface, and the simplified 2D case, in which the scene is modeled as a curve and Equation (4) becomes a contour integral.

### 2.4. Inversion

As previously demonstrated, the aim of our inversion is to minimize the discrepancy between preprocessed radar data and their simulated counterpart (evolving shape) and, by carrying this out, to obtain an evolving shape that matches the actual scene.

It is important to understand that a radar echo contains range information (i.e., distance from the antennas). This is the reason why point scatterers located in the same range contribute similarly to the signal (same frequency signature in the deramped signal) regardless of the actual location. Therefore, there is a natural directional ambiguity that can be solved by collecting data from different view points, i.e., with multiple transmitter–receivers or with a moving antenna pair.

Since we have a discretized range profile, the fidelity term of our cost functional will use the vector of the average electric field strength values of the range bins for a given antenna. For the *j*-th antenna pair, we denote this vector with $H_{j}$. Based on this, the data fidelity term is given as follows:

(5) $$E_{D}=\frac{1}{2}\sum_{j=1}^{N_{a}}{\left[D_{j}^{T}\left(H_{j}-H_{j}^{0}\right)\right]}^{2}$$

where $N_{a}$ is the number of antenna pairs, $H_{j}$ is the simulated radar response computed using Equation (4), and $H_{j}^{0}$ is the observable extracted from the measured data (preprocessed). The vector $D_{j}$ consists of the average range values of the range bins, which are used to compensate for the range attenuation of the signal strength over the range so that each bin contributes to the error functional similarly.

**Figure 4:**
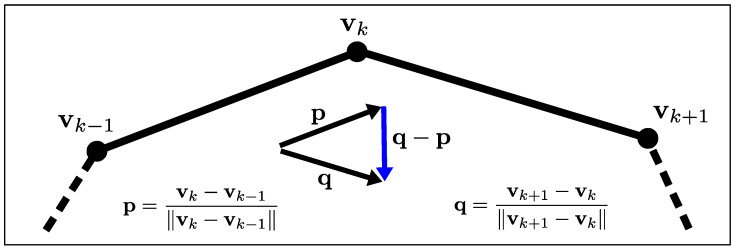
Illustration of our curvature-based regularizer implemented for a polygonal object. For a control point connecting two adjacent edges, this regularizer imposes a penalty on the magnitude of the vector derived from the difference between the directions of these edges, thereby favoring shape updates that progressively enhance geometric smoothness.

Regarding the regularization term, we have considerable flexibility in incorporating various shape priors. For the scope of this work, we define it as the shape’s curvature, which naturally promotes smooth geometries. The parameterization selected for the shape becomes crucial at this stage, as we must customize our curvature regularizer to accommodate this specific representation. For ease of implementation, we focus on 2D star-shaped objects. This class of geometries offers dual advantages: They can be readily approximated using polygonal shapes (i.e., sets of interconnected segments) and can be represented with particular elegance via polar coordinates. This approach simplifies curve discretization, allowing us to treat the shape as a polygonal entity and transform the shape optimization problem into one of optimizing the radial positions of the vertex points (or control points) that define the shape relative to a predetermined origin. Our regularizer (illustrated in Figure 4) is consequently expressed as follows:

(6) $$E_{R}=\frac{1}{2}\sum_{k=1}^{N_{v}}{∥\frac{v_{k+1}-v_{k}}{∥v_{k+1}-v_{k}∥}-\frac{v_{k}-v_{k-1}}{∥v_{k}-v_{k-1}∥}∥}^{2}$$

where $N_{v}$ is the number of vertices ($v_{-1}=v_{N_{v}}$ and $v_{N_{v}+1}=v_{1}$ because of the circular topology of the shape). By combining Equations (5) and (6), we formulate our complete energy functional as follows:

(7) $$E=E_{D}+\lambda E_{R}$$

where $E_{D}$ promotes reductions in the disparity between the evolving shape and the actual shape (data fidelity), while $E_{R}$ encourages smoothness in the evolving shape, with λ serving as the regularization weight factor.

### 2.5. Optimization

To perform the iterative evolution of the scene, we calculate the gradient of the energy functional in Equation (7) with respect to our chosen parameterization, specifically the coordinates of the points of the vertex.

Each vertex determines the location of the two edges directly connected to it. Considering one pair of antennas, displacing either of the two edges will correspondingly affect the radar’s response provided that such edges are visible to both the transmitter and receiver. In particular, such perturbations will affect the range bins that include the affected edges. As such, for every antenna pair configuration and every associated range bin, we need to find out which edges, or portions of edges, fall within the range span of the bin and whether such segments are visible. This task is relatively simple in 2D and can be achieved using Algorithm 1.
**Algorithm 1** Visibility analysis for the edge of the polygonal shape model. $e_{k}^{k+1}$ denotes the edge connecting $v_{k}$ to $v_{k+1}$.$S^{c}\leftarrow \left\{\right\}$ **for** $e_{i}^{i+1}\in \left\{e_{1}^{2},e_{2}^{3}\cdots ,e_{N_{v}}^{1}\right\}$**do**
   $p_{i}^{c}\leftarrow$ midpoint of $e_{i}^{i+1}$
   $p_{i}^{a}\leftarrow$ antenna pair location
   $l_{i}\leftarrow$ hypothetical line segment connecting $p_{i}^{c}$ to $p_{i}^{a}$
   **for** $e_{j}^{j+1}\in \left\{e_{1}^{2},e_{2}^{3}\ldots ,e_{N_{v}}^{1}\right\}\setminus e_{i}^{i+1}$ **do**
     **if** $l_{i}$ intersects $e_{j}^{j+1}$ **then**
        $S^{c}\leftarrow S^{c}+e_{i}^{i+1}$
        **break**
     **end if**
   **end for**
**end for**
**return**
$\left\{e_{1}^{2},e_{2}^{3}\ldots ,e_{N_{v}}^{1}\right\}\setminus S^{c}$


The pseudocode (Algorithm 1) accepts a list of input control nodes *n* and returns those visible from the transmitter and receiver pair of interest. This algorithm has a complexity of $O(n^{2})$, which we chose due to its ease of implementation. Note that there are other alternatives that are more computationally efficient and can be used when moving toward 3D applications [24,25,26].

**Figure 5:**
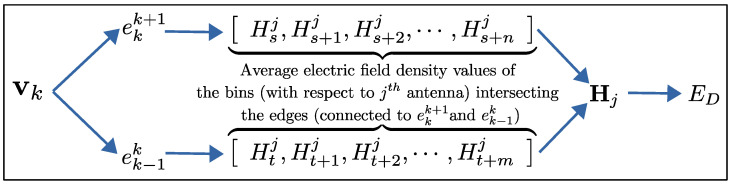
Dependency graph demonstrating the relationship between vertex coordinates and $E_{D}$. For edges (*e*), subscripts and superscripts indicate the connected vertices. For average electric field strengths, subscripts represent range bin indices while superscripts denote antenna indices.

After establishing visibility, we derive the energy gradient using the chain rule. Figure 5 illustrates the relationship between the energy functional data fidelity component ($E_{D}$) and the coordinates of a vertex $v_{k}$.

**Figure 6:**
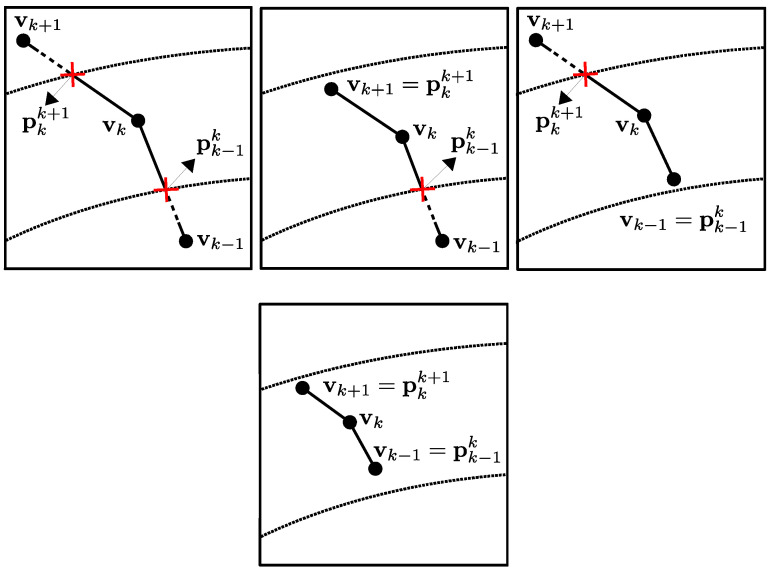
Possible placements of vertices with respect to a range bin when vertex $v_{k}$ is included in the *i*-th range bin.

Although it is relatively straightforward to calculate the partial derivative of $E_{D}$ with respect to $H_{j}$ and that of $H_{j}$ with respect to $H_{i}^{j}$, determining the partial derivatives of $H_{i}^{j}$ with respect to the location of the vertex $v_{k}$ requires particular attention. This complexity arises because edges may span multiple range bins, causing integration boundaries to depend on polygon vertices where the connected edge intersects with the range boundaries. Figure 6 shows several possible configurations of this relationship. The expression for the partial derivative of $H_{i}^{j}$ with respect to the *k*-th vertex is given as follows:

(8) $$\frac{\partial H_{i}^{j}}{\partial v_{k}}=\frac{\partial }{\partial v_{k}}\left(\int_{S_{i}^{j}\cap e_{k-1}^{k}}Q\left(x,n\right)\mathrm{ds}+\int_{S_{i}^{j}\cap e_{k}^{k+1}}Q\left(x,n\right)\mathrm{ds}\right).$$

Here, $S_{i}^{j}$ represents the set of shape boundary points contained within the *i*-th range bin relative to the *j*-th antenna pair. The domain of integration becomes the portion of the edge contained within the range bin. Taking the derivative of this expression yields the following:

(9) $$\frac{\partial H_{i}^{j}}{\partial v_{k}}=\int_{S_{i}^{j}\cap e_{k-1}^{k}}\frac{\partial }{\partial v_{k}}Q\left(x,n\right)\mathrm{ds}+\int_{S_{i}^{j}\cap e_{k}^{k+1}}\frac{\partial }{\partial v_{k}}Q\left(x,n\right)\mathrm{ds}+B_{up}+B_{lo}$$

where $B_{up}$ and $B_{lo}$ are boundary terms that take the following form:

(10) $$\begin{array}{ccc} B_{up} & = & Q\left(p_{k}^{k+1},n\right)\frac{\partial ∥p_{k}^{k+1}-v_{k}∥}{\partial p_{k}^{k+1}}\frac{\partial p_{k}^{k+1}}{\partial v_{k}},\;\mathrm{for}\;p_{k}^{k+1}\neq v_{k+1} \end{array}$$

(11) $$\begin{array}{ccc} B_{lo} & = & Q\left(p_{k-1}^{k},n\right)\frac{\partial ∥v_{k}-p_{k}^{k-1}∥}{\partial p_{k-1}^{k}}\frac{\partial p_{k-1}^{k}}{\partial v_{k}},\;\mathrm{for}\;p_{k-1}^{k}\neq v_{k-1}. \end{array}$$

We have $B_{up}=0$ when the edge connecting $v_{k+1}$ to $v_{k}$ is fully contained in a single range bin, and similarly, we have $B_{lo}=0$ when the edge connecting $v_{k}$ and $v_{k-1}$ is contained in a single range bin. In these cases, the dependency of the integration domain on $v_{k}$ vanishes. In summary, our selection of star-shaped objects allows us to utilize a polar representation in which control nodes maintain fixed angular positions while only their radial distances vary. Consequently, we adopt the radii of the vertices as our parameter set for shape evolution. Our gradient vector incorporates the component of the derivative of the energy functional with respect to the vertex coordinates along the radial direction. For the *k*-th vertex, this gradient component is expressed as follows:

(12) $$\frac{\partial E}{\partial \phi_{k}}=\langle \frac{\partial E}{\partial v_{k}},\frac{v_{k}}{∥v_{k}∥}\rangle .$$

Finally, the shape update is performed by perturbing the nodes in the direction opposite to this gradient. As a final remark, the rationale behind our choice of data preprocessing was to address the oscillatory nature of radar signals and obtain a well-behaved cost functional for inversion. However, several shallow local minima can still persist in the energy landscape. Therefore, to help avoid these shallow minima during optimization and improve the convergence rate, we improved gradient descent by adopting the accelerated descent algorithm in [27], resulting in the following updated scheme:

(13) $$V_{i+1}=\alpha V_{i}-\beta \frac{\partial E}{\partial \Phi }\Phi_{i+1}=\Phi_{i}+V_{i+1}$$

where V represents the velocity; Φ denotes the radii of the vertices being updated; α is the momentum coefficient; β indicates the step size of the gradient descent.

## 3. Results

To evaluate the effectiveness of the algorithm, we conducted simulations in three distinct scenarios, each involving multiple pairs of antennas. Since we are working with a 2D case, we assume that both the transmitter (TX) and receiver (RX) antenna apertures have geometries consisting of side-by-side line segments sharing the same unit normal. Table 1 presents several parameters that remained constant throughout all simulations.

The antenna gain terms are assumed as follows:

(14) $$\begin{array}{ccc} G^{\prime } & = & A_{x^{\prime }}\sqrt{\cos\left(\theta^{\prime }\right)}sinc\left(\frac{fA_{x^{\prime }}\sin\left(\theta^{\prime }\right)}{c}\right) \end{array}$$

(15) $$\begin{array}{ccc} G & = & A_{x}\sqrt{\cos\left(\theta \right)}sinc\left(\frac{fA_{x}\sin\left(\theta \right)}{c}\right). \end{array}$$

**Figure 7:**
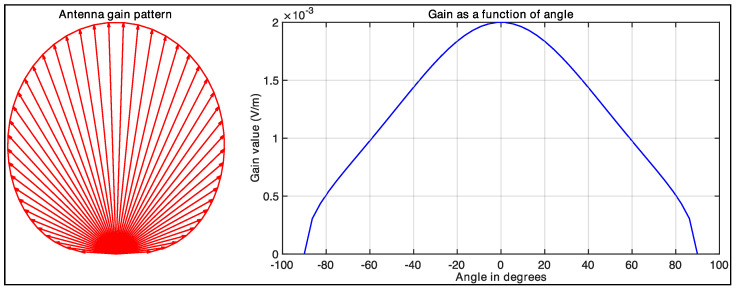
Illustration of the antenna gain pattern’s polar plot (left) and gain value over the angle (right).

In these expressions, $A_{x}$ and $A_{x^{\prime }}$ represent the aperture lengths for the transmitter and receiver antennas, respectively. Angles θ and $\theta^{\prime }$ denote the angles formed by the transmitted and received rays relative to the antenna’s surface normal, while *c* represents the speed of light. Using the parameters specified in Table 1, we compute the antenna gain pattern and plotted the gain values as a function of angle, as shown in Figure 7.

For each simulation, we used different shape configurations (consisting of an initial object and an actual object) and tested two distinct antenna arrangements for each configuration. In our first setup, we utilized a circular antenna array where each antenna pair was oriented toward the center of the array, ensuring that at least one antenna pair could detect each portion of the target object. Our second setup featured antennas arranged in a linear array, mimicking configurations more commonly found in traditional radar applications. Throughout each experiment, we initially applied a high regularization coefficient to maintain shape regularity; then, we gradually reduced this coefficient to progressively capture increasingly finer details. Table 2 provides a summary of the shapes used in our experiments.

### 3.1. Circular Array of Antennas

**Figure 8:**
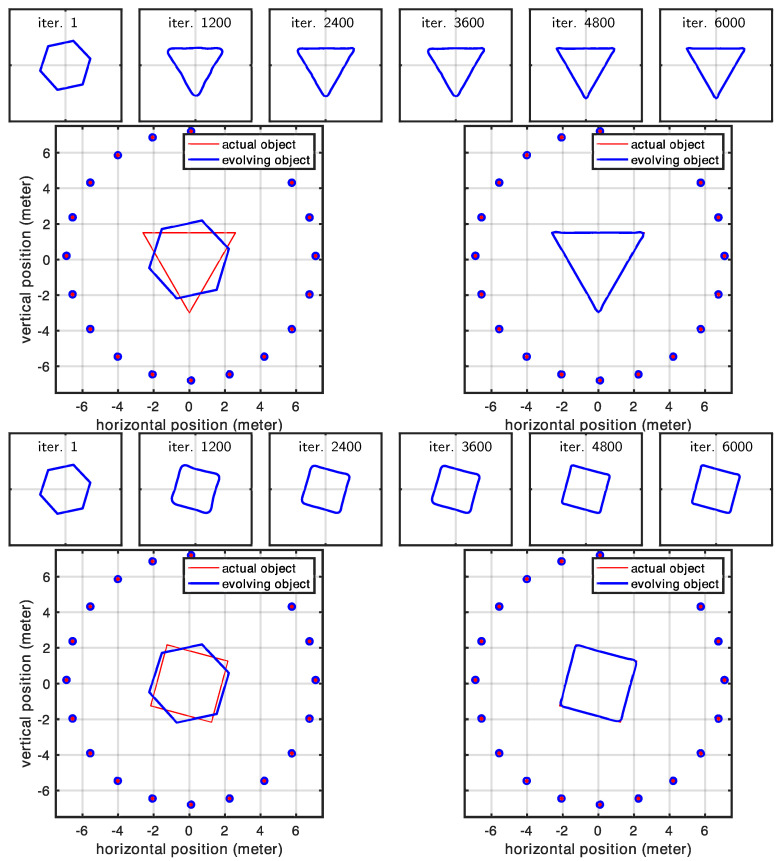
Evolution of the shape model for Case 1 and Case 2 in a circular array of antennas.

**Figure 9:**
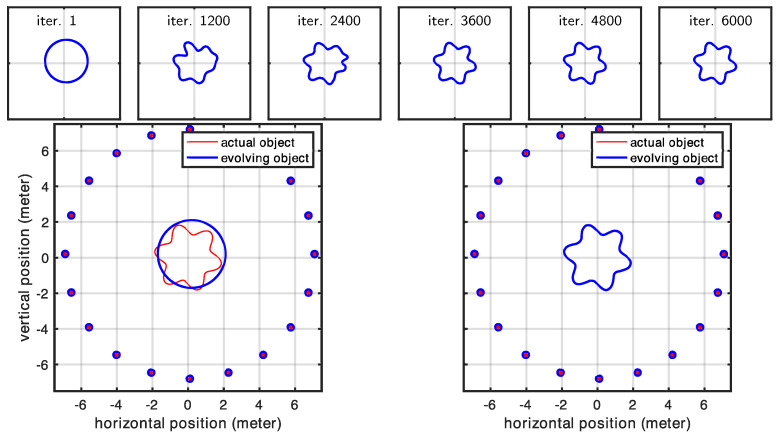
Evolution of the shape model for Case 3 in the case of a circular array of antennas.

**Figure 10:**
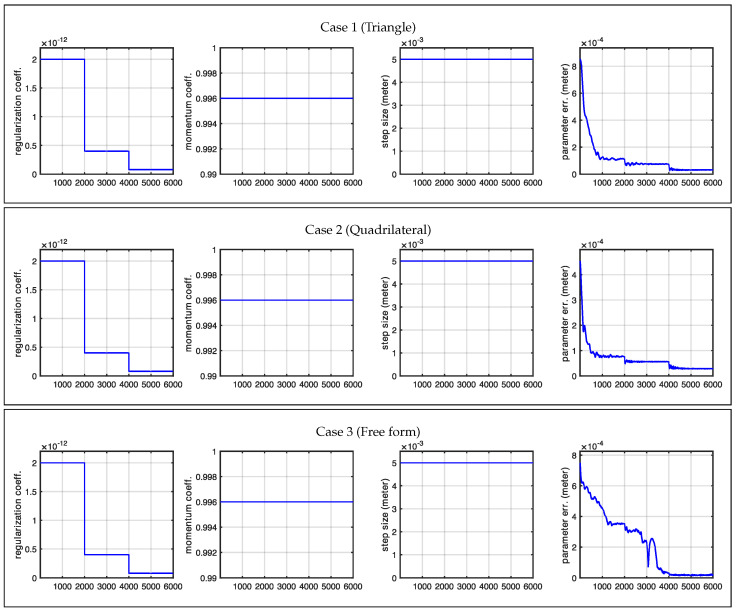
Optimization plots for Case 1, Case 2, and Case 3 for a circular antenna pattern across iterations.

In this experiment, we examined a shape surrounded by antennas positioned to ensure that each portion of the investigated shape was visible to at least one pair of antennas. This arrangement led us to expect that our initial shape estimate would properly converge to the actual shape. Our expectations were largely met with impressive high-quality reconstruction results. The only exceptions occurred at the very sharp corners in Cases 1 and 2 (in Figure 8). However, these minor discrepancies were entirely expected, as the regularization term naturally favors smooth shapes and suppresses high-curvature details, causing the corners of the evolving model to appear rounded. In contrast, Case 3 (in Figure 9), where the true shape had no sharp corners, showed correctly retrieved contours throughout the entire shape. Figure 10 shows the statistics related to this experiment. In particular, the figure shows the changes we made in the regularization coefficient, which was progressively lowered to reduce the effect of the regularization term, and the cumulative distances of the shape control points from the desired location (true locations). A rough approximation of the desired shape was reached after 2000 iterations, while full convergence required 6000 iterations. The total computational time was less than two minutes for each case.

### 3.2. Linear Array of Antennas

**Figure 11:**
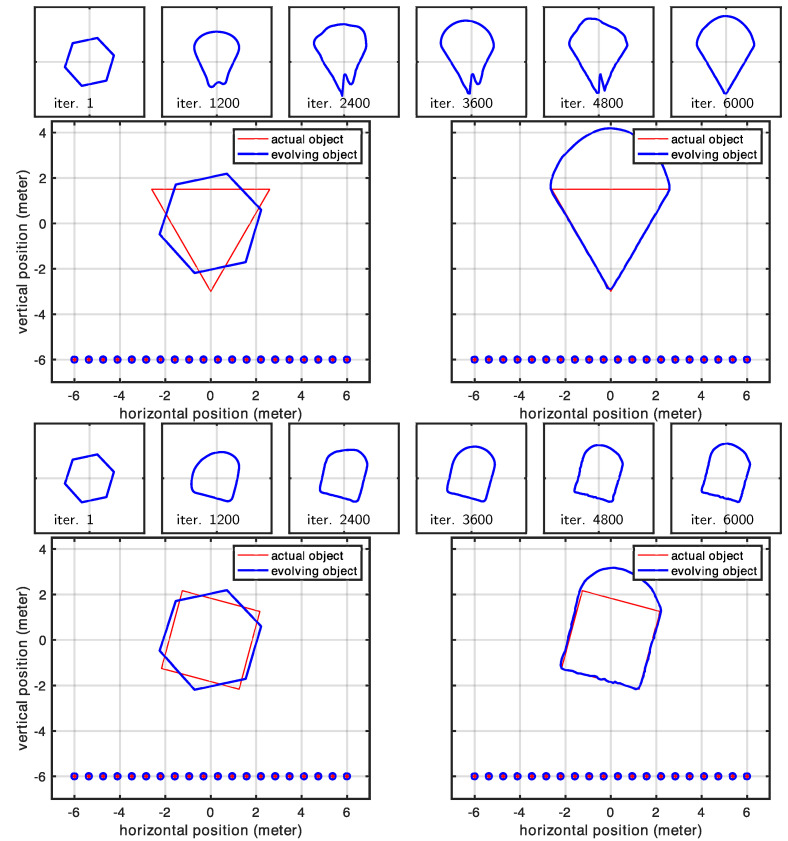
Evolution of the shape model for Case 1 and Case 2 in the case of a linear antenna pattern.

**Figure 12:**
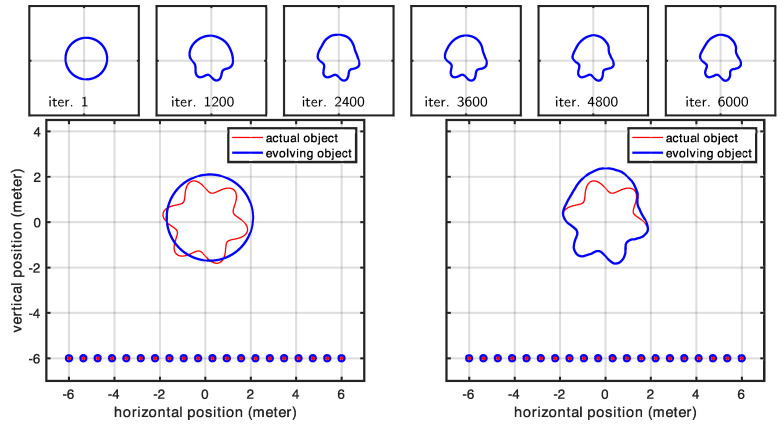
Evolution of the shape model for Case 3 in the case of a linear antenna pattern.

**Figure 13:**
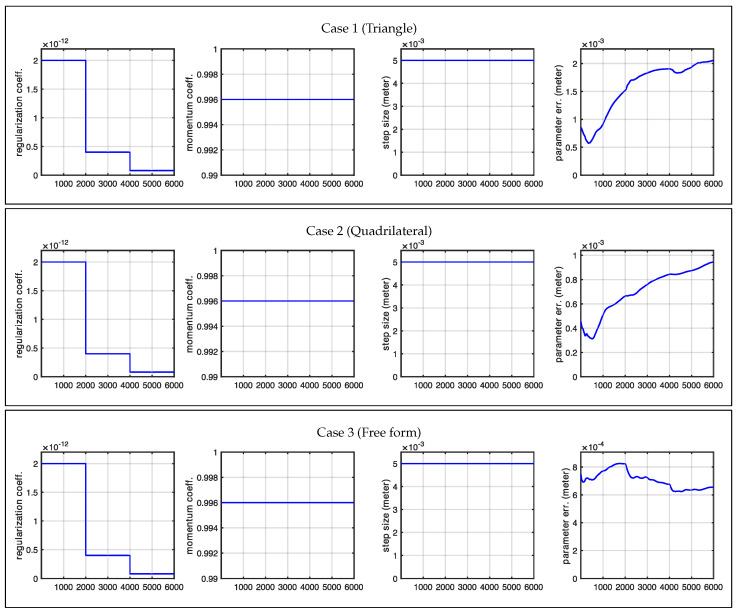
Optimization plots for Case 1, Case 2, and Case 3 for a linear antenna pattern across iterations.

In this experiment, we investigated a linear antenna array configuration. Since the shape was illuminated from only one side, portions of the scene contour remained invisible and therefore could not be reconstructed. Our results confirmed this expected behavior while also highlighting the effect of the regularizer, which served as the only force driving the evolution of the invisible parts of the contour. In contrast, the contour facing the antenna array was perfectly reconstructed, as shown in Figure 11 and Figure 12. The shaded part of the scene, evolving solely according to the regularizing term, was also responsible for the increased parameter error observed in all cases (see Figure 13). We should emphasize that although the dominance of the regularizer initially appears to create an undesired artifact (the curved boundary), this behavior is intentional by design. In practice, since we know exactly which portions of the contour are illuminated and which are not, we can precisely identify which parts of the scene are correctly retrieved and expected to match the actual shape. Figure 13 shows the statistics related to this second experiment. Similarly to Section 3.1, full convergence required 6000 iterations, where each case took less than 2 min to complete. The larger error observed here is due to the part of the shape that was invisible to the antennas, which could not be recovered.

### 3.3. Shape Parametrization Using Active Contours and the Level-Set Method

Note that the continuous variational framework developed here is not limited to any particular class of discrete geometric representation. In our work with respect to the time domain, we began from a graph-based representation and subsequently generalized our algorithm to 3D applications. Similarly, we decided to provide a proof of concept in 2D before generalizing it to 3D. However, the graph-based approach poses several limitations. Therefore, while we restricted this investigation to 2D for simplicity, we nevertheless selected a flexible finite element representation that is capable of representing objects both in the forms of a graph and compact objects. Additionally, the present approach can be easily reformulated in 3D. Although moving from 3D to 2D simulations seems to represent a step backward, it nonetheless demonstrates greater geometric flexibility compared to the graph representations used in our early work. Ultimately, we seek a fully 3D method that has the flexibility to represent a broad class of geometries, including compact surfaces, graphs, multiple separate surfaces, and surfaces of complex topology (with holes). For these reasons, implicit representations (i.e., level-set methods) offer a much better choice in 3D.

However, the additional details needed to develop and implement a 3D level-set method are beyond the scope of this paper, but they will indeed be the focus of an upcoming paper dedicated to fully developing the level-set framework for this radar inversion strategy. However, we do take a first step in this direction by including a 2D level-set example in our experimental results, which demonstrates another level of flexibility. While the 2D finite element approach gives us the ability to capture a prior-known number of compact shapes, the 2D level set extends this to a prior unknown number by allowing contour splitting and merging. We have omitted the implementation details here for the 2D level-set method, as the same (and many more) details will, by necessity, be covered with the fully 3D method that is being developed for publication.

**Figure 14:**
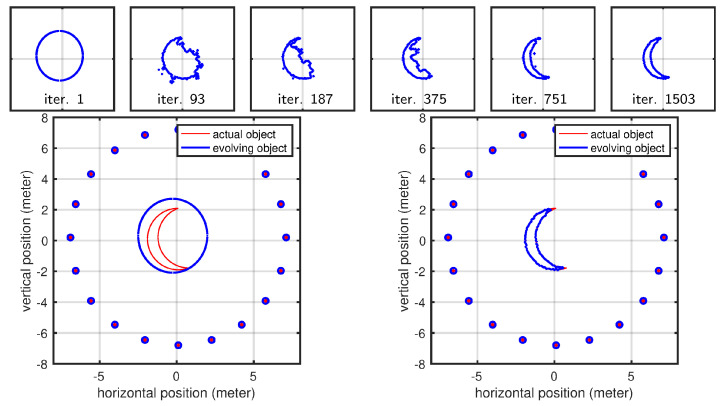
Evolution of the shape model for a crescent shaped object.

**Figure 15:**
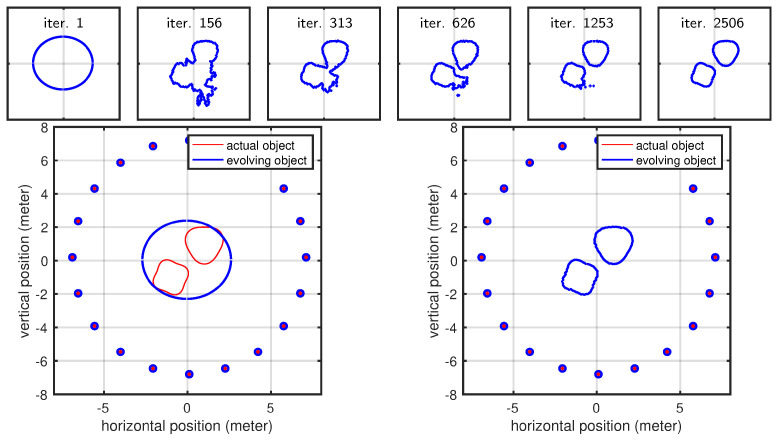
Evolution plots for the second case in which we showcase a topological change that is naturally handled by LSM.

Here, we demonstrate the level-set approach using two different cases that cannot be handled by our polygonal shape model: (1) a crescent shape that cannot be expressed as a polygonal shape used for other results (we parameterized the polygonal shape model using a polar representation, and for a crescent-shaped object, the same angle value corresponds to multiple radii, meaning that a ray emanating from the center can intersect with the shape at multiple points) and (2) a geometric shape that is topologically different compared to the initial shape mode (level-set parameterization helps us here since we do not have to assume a specific topology as our initial shape model). The evolution of the curve for the crescent-shaped object is shown in Figure 14. The curve evolution of the second case is shown in Figure 15. The present section anticipates early results using active contours and the level-set method (LSM). The motivation for using these methodologies to parameterize the scene is twofold: (1) LSM describes the scene implicitly, with greater flexibility (and simplicity) in representing any shape virtually; (2) it naturally handles topological changes. We reserve the full discussion of such approaches for a subsequent article, in which we will address the problem in the full three-dimensional case, which is more complex but also more relevant for real-world applications. Nevertheless, we include this early result in 2D to demonstrate that this geometric approach can indeed be used in the context of radar-based inversion.

## 4. Conclusions and Future Work

We propose a frequency-domain radar-based shape inversion method that is analogous, yet complementary, to the time-domain approach described in [15,16]. Our 2D inversion examples demonstrate that stretch-processed radar signals can effectively be used to reconstruct scene shapes when proper observables are extracted from them. Unlike classical radar imaging techniques that rely solely on signal processing, our approach leverages data inversion using an explicit geometric model that attempts to capture the scene shape by iteratively minimizing a cost functional. This modeling approach allows us to incorporate various shape priors directly into the estimation process. In this paper, we specifically implemented a regularization term that promotes smooth shapes. Nevertheless, we should emphasize that any geometric shape prior knowledge could be integrated naturally as a regularization term in our cost functional. Another important benefit of using geometric models is their ability to handle visibility issues and occlusions effectively. Our approach implements visibility analysis to identify which segment of objects are detectable by radar antennas and thus influence the received signal’s measurements. We observe the effects of the visibility analysis in Section 3.2, where the visible parts of the scene successfully converge to the true shape while the occluded parts evolve toward a configuration that satisfies the mathematical prior built into the regularizer. The latter is therefore an artifact. However, this is a design choice. Since we explicitly computed visibility, we can discern exactly which part of the scene is correctly reconstructed.

Although the current experiments used the parameterization of the scene relative to polar coordinates and investigated polynomial shapes, such assumptions do not impact the validity of our findings, which carry over to more complex scene representations. Similarly to our early work in the time domain, we provide a proof of concept in a simplified environment. However, in contrast to pursuing a graph-based representation of the geometry (which posed several limitations), we instead opted for a more flexible shape based on finite elements. Although implemented in 2D for simplicity, the chosen representation not only removes the limitations of the graph-based approach but also provides us with a flexible environment to test the viability of the method without the burden of implementing the approach directly into 3D.

It should be noted that even though our method was implemented in C++17 (both forward and inverse models and visibility analysis), our main focus was on correctness and simplicity (e.g., the visibility analysis has computational complexity $O(n^{2})$) and not performance. Therefore, considering the fact that numeric integration is the computation-heavy part of our method and these computations are known to significantly benefit from parallel programming and vector extensions available in modern processors, we can comfortably say that there is significant room for improvement in future work. While, in previous work, we demonstrated that radar-based shape reconstruction is feasible in the time domain, the present experiments, although exploring the simplified case of two-dimensional geometries, nevertheless demonstrate that the same shape reconstruction framework can also be formulated in the frequency domain. This conclusion motivates us to pursue the challenge of implementing the approach using active surfaces, as these methods provide the flexibility necessary to handle any topological change (i.e., evolving an initial contour to reconstruct multiple separated objects within the same scene). However, such an effort is a matter for future work.

## Figures and Tables

**Table 1:** Simulation configuration.

Number of antenna pairs	20
Aperture width	2 mm
Antenna pair offset (between TX and RX)	6 mm
Carrier center frequency	79 GHz
Signal bandwidth	4 GHz
Chirp rate	4 × $10^{13}$ Hz/s
Number of range bins	50
Minimum/maximum range	2/21 m
Number of vertices of the evolving shape	120

**Table 2:** Different shape configurations used for simulations.

Name	Actual Shape	Initial Shape
Case 1	Triangle	Hexagon
Case 2	Quadrilateral	Hexagon
Case 3	Free form	Circle

## Data Availability

The original contributions presented in this study are included in the article. Further inquiries can be directed to the corresponding author.

## References

[1] Yezzi A., Soatto S. (2003). Stereoscopic segmentation. Int. J. Comput. Vis..

[2] Richards M.A., Scheer J., Holm W.A. (2010). Principles of Modern Radar.

[3] Brown W.M. (1967). Synthetic aperture radar. IEEE Trans. Aerosp. Electron. Syst..

[4] Carrara W.G., Goodman R.S., Majewski R.M. (1995). Spotlight Synthetic Aperture Radar.

[5] Cheney M. (2001). A mathematical tutorial on synthetic aperture radar. SIAM Rev..

[6] Massonnet D., Souyris J.C. (2008). Imaging with Synthetic Aperture Radar.

[7] Massonnet D., Rossi M., Carmona C., Adragna F., Peltzer G., Feigl K., Rabaute T. (1993). The displacement field of the Landers earthquake mapped by radar interferometry. Nature.

[8] Jakowatz C., Thompson P. (1992). The Tomographic Formulation of Spotlight Mode Synthetic Aperture Radar Extended to Three Dimensional Targets.

[9] Jakowatz C.V., Thompson P. (1995). A new look at spotlight mode synthetic aperture radar as tomography: Imaging 3-D targets. IEEE Trans. Image Process..

[10] Lopez-Sanchez J.M., Fortuny-Guasch J. (2000). 3-D radar imaging using range migration techniques. IEEE Trans. Antennas Propag..

[11] Virieux J., Operto S. (2009). An overview of full-waveform inversion in exploration geophysics. Geophysics.

[12] Goncharsky A.V., Romanov S.Y. (2011). On a problem of ultrasonic tomography. Numer. Methods Program..

[13] Seidl R., Rank E. (2017). Full waveform inversion for ultrasonic flaw identification. AIP Conf. Proc..

[14] Yildirim A., Yezzi A. Developing a Geometric Deformable Model for Radar Shape Inversion. Proceedings of the 2018 IEEE International Conference on Acoustics, Speech and Signal Processing (ICASSP 2018).

[15] Bignardi S., Yezzi A.J., Yildirim A., Barnes C.F., Sandhu R. (2021). A Feasibility Study of Radar-Based Shape and Reflectivity Reconstruction Using Variational Methods. Inverse Probl..

[16] Bignardi S., Sandhu R., Yezzi A. (2023). Radar-Based Shape and Reflectivity Reconstruction Using Active Surfaces and the Level Set Method. IEEE Trans. Pattern Anal. Mach. Intell..

[17] Ma Y., Soatto S., Kosecka J., Sastry S.S. (2012). An Invitation to 3-D Vision: From Images to Geometric Models.

[18] Horn B., Klaus B., Horn P. (1986). Robot Vision.

[19] Budge M.C., German S.R. (2015). Basic RADAR Analysis.

[20] Caputi W.J. (1971). Stretch: A time-transformation technique. IEEE Trans. Aerosp. Electron. Syst..

[21] Yildirim A. (2020). A Geometric Variational Approach To Shape Inversion For Radar. Ph.D. Thesis.

[22] Jakowatz C.V., Wahl D.E., Eichel P.H., Ghiglia D.C., Thompson P.A. (2012). Spotlight-Mode Synthetic Aperture Radar: A Signal Processing Approach: A Signal Processing Approach.

[23] Goodman W.G.C.R.S., Majewski R.M. (1995). Spotlight Synthetic Aperture Radar: Signal Processing Algorithms.

[24] Foley J.D., Van F.D., Van Dam A., Feiner S.K., Hughes J.F., Hughes J., Angel E. (1996). Computer Graphics: Principles and Practice.

[25] Stewart A.J., Karkanis T. (1998). Computing the approximate visibility map, with applications to form factors and discontinuity meshing. Rendering Techniques’ 98.

[26] Coorg S., Teller S. Real-Time Occlusion Culling for Models with Large Occluders. Proceedings of the 1997 ACM Symposium on Interactive 3D Graphics.

[27] Nesterov Y. (1983). A method of solving a convex programming problem with convergence rate O (1/k2). Sov. Math. Dokl..

